# Socioeconomic inequalities in physical function decline: multilevel longitudinal results from the HABITAT study

**DOI:** 10.1186/s12889-025-23309-8

**Published:** 2025-07-03

**Authors:** Jerome N Rachele, Venurs Loh, Anna Timperio, Jenny Veitch, Rees Thomas, Rebecca A Reid, Wendy J Brown

**Affiliations:** 1 College of Sport, Health, and Engineering, Victoria University, Melbourne, Australia; 2 Institute for Health and Sport, Victoria University, Melbourne, Australia; 3 Institute for Physical Activity and Nutrition, School of Exercise and Nutrition Sciences, Deakin University, Geelong, Australia; 4 Faculty of Health Sciences and Medicine, Bond University, Gold Coast, Australia; 5 School of Human Movement and Nutrition Sciences, The University of Queensland, Brisbane, Australia

**Keywords:** Physical function, Ageing, Social inequalities, Neighbourhood, Built environment

## Abstract

**Background:**

Australia’s population is ageing, with a projected continued increase in the proportion of individuals aged 65 years and older. Good physical function is important to ensure independence and mobility among older adults. This study examined changes in physical function by socioeconomic indicators including education, occupation, household income and neighbourhood socioeconomic disadvantage.

**Methods:**

Data were from waves four (2013) and five (2016) (1,186 men and 1,673 women) of the HABITAT study, a multilevel longitudinal study of adults aged 40–65 at baseline (2007) living in 200 neighbourhoods in Brisbane, Australia. Individual-level socioeconomic indicators were self-reported and physical function was self-reported using the 10-item subscale of the Short-Form 36 survey, with scores ranging from 0 to 100. Neighbourhood socioeconomic disadvantage was obtained from a census-based Index of Relative Socioeconomic Disadvantage score. Data were analysed using multilevel linear regression.

**Results:**

Pooled analysis showed graded inequalities in physical function across all socioeconomic groups: those with lower levels of education, occupation and household income all had lower function, while residents of the most disadvantaged neighbourhoods had 8.16 lower function (95%CI: 10.21, 6.12) than those in the most advantaged neighbourhoods. Over the three-year period, there was a mean reduction in physical function scores of 1.97 (95%CI: -2.58, -1.36), though physical function inequalities did not widen over time between socioeconomic groups.

**Conclusion:**

There was little evidence of inequalities in the magnitude of decline in physical function across socioeconomic groups between the two time points. Future research should consider more objective performance-based measures to better understand the complexity of physical function among the ageing population.

## Introduction

Australia’s population is ageing [1]. Data derived from the Australian Bureau of Statistics (ABS) reveals a continued rise in the proportion of individuals aged 65 and above [2]. By 2050, the number of people aged 60 years and above is projected to reach two billion globally, which is 185% higher than in 2019, and will account for 22% of the world’s population [3]. The ageing population reflects'a triumph of humanity, but also a challenge to society' [4]. While people in the 21st Century are living much longer than in previous eras due to improved living standards (e.g., safer and cleaner housing, sanitation and water quality) and medical advancement, there are concerns about the capacity to maximise quality of life and ensure that any additional years are free from disability [5].

Physical function is defined as the ability to undertake everyday activities that require physical capacity [6]. Optimising physical function as people age is critical for undertaking everyday activities like shopping, completing household chores, and engaging in recreational pursuits, and contributes to overall quality of life. Strong evidence supports the role of physical activity in improving and maintaining physical function, slowing the rate of decline, and enhancing independence in later life [7]. On the other hand, declining physical function is a precursor of disability. Empirical literature has established a link between age and physical function; while the onset of decline in function begins in mid-life with more rapid declines in later-life, the rate of decline varies by individual- and environmental-level factors [5, 8].

Socioeconomic inequalities, at an individual or area-level, exist across health behaviours and outcomes. At an individual level, socioeconomic indicators, in particular educational attainment and income, have been shown to influence physical function. Individuals with lower education may have limited psychological resources to make health care and lifestyle decisions, resulting in a steeper decline in physical function [9]. Similarly, at an area-level, residing in a disadvantaged neighbourhood, that is, socioeconomic conditions of an area that may adversely affect the health and wellbeing of its residents, including collective poverty, unemployment, lower education levels, and poor access to services and amenities within the neighbourhood [10], may contribute to a more rapid decline in physical function compared to residing in less disadvantaged neighbourhoods. Residents of disadvantaged neighbourhoods have reported higher levels of perceived neighbourhood stressors, reduced social cohesion, lower perceived safety from crime, and poor aesthetics (e.g., graffiti, vandalism) [11], all of which are associated with lower physical functioning [12]. These neighbourhood characteristics may hinder health promoting behaviours, such as physical activity, which plays a pivotal role in physical function [13].

While there are several cross-sectional multilevel studies demonstrating associations between neighbourhood disadvantage and physical function [15–17], the few longitudinal studies that have explored this relationship have reported mixed findings. In Australia, a study that followed women for 14–16 years found that those living in the most disadvantaged neighbourhoods had the lowest physical function score, compared with those living in the most advantaged neighbourhoods and that those living in the most disadvantaged neighbourhoods had the steepest decline in physical function from baseline to follow-up [8]. Similarly, a prospective cohort study of older adults in the UK aged ≥ 60 years with a 2-year follow-up found those living in the most disadvantaged neighbourhoods had a higher risk of incident mobility difficulty (i.e. difficulty performing basic daily function such as walking and climbing) and impaired gait speed over the 2-year period than those living in the most advantaged neighbourhoods [18]. Conversely, a longitudinal US study conducted among men and women aged 55–65 years at baseline found that neighbourhood disadvantage did not predict the onset of decline in physical function at 10-year follow up [19]. The inconsistent findings between these longitudinal studies could be due to differences in geographical location, follow-up periods, measurement, and analytical approaches. Of note, these studies did not account for neighbourhood self-selection (i.e., people choosing where they live to suit or support their lifestyle), which may confound the relationship between neighbourhood disadvantage and physical function [20]. Research of this nature is particularly important for Australian cities, where there is clear spatial segregation of socioeconomic disadvantage, with disadvantaged neighbourhoods often experiencing poorer access to health-promoting resources such as green space, transport, and healthcare services [21]. These area-level disparities may contribute to accelerated declines in physical function among residents of disadvantaged neighbourhoods.

Inconsistent longitudinal findings in previous research highlights the need for greater understanding of the links between socioeconomic indicators and physical function decline. Considering the ageing population globally and consequent burden to health care systems, it is important to examine socioeconomic trends in physical function inequalities to identify opportunities to improve health related behaviour and reduce the rate of physical function decline. This study aimed to examine trends in physical function over time in a population-representative sample of mid-older aged adults, and further examine whether these trends differed according to individual- and neighbourhood-level socioeconomic indicators.

## Methods

Data from the HABITAT (**H**ow **A**reas in **B**risbane **I**nfluence Heal**T**h and **A**c**T**ivity) study were used for this investigation. HABITAT is a multilevel longitudinal study designed to examine the relative contributions of environmental, social, psychological and sociodemographic factors on the patterns and change in physical activity and health from 2007 to 2016 among mid-older aged men and women living in the Brisbane Local Government Area, Australia [22]. The HABITAT study received ethical clearance from the RMIT University Human Research Ethics Committee (CHEAN B 20577-01/17), in alignment with the principles outlined in the Declaration of Helsinki. All participants in this study were informed about the study’s objectives, procedures, potential risks, and benefits, and their rights to withdraw at any time without any consequences, and provided informed consent prior to their inclusion.

### Sample

Details about HABITAT’s baseline sampling have been described elsewhere [23]. A multi-stage probability sampling design was used to select a stratified random sample of adults aged 40–65 years from 200 Census Collector’s Districts (CCD) - the smallest administrative unit used by the Australian Bureau of Statistics to collect census data. A mean of 85 people were recruited from within each CCD. CCDs are embedded within a larger suburb, hence the area corresponding to, and immediately surrounding, a CCD is likely to have meaningful influence on the residents. For this reason, we use the term ‘neighbourhood’ to refer to each CCD. The baseline HABITAT sample in 2007 was broadly representative of the wider Brisbane population of adults aged 40–65 years [24].

### Data collection and response rates

A structured self-administered questionnaire was developed that asked respondents about their neighbourhood, participation in physical activity, correlates of activity, health and well-being; and sociodemographic characteristics. The questionnaire was sent during May-July in 2007, 2009, 2011, 2013 and 2016 using the Dillman [25] mail survey method. After excluding out-of-scope respondents (i.e., deceased, no longer at the address, unable to participate for health-related reasons), the total number of usable surveys returned in each survey wave was 11,035 (68.3%), 7866 (72.3%), 6900 (66.7%), 6520 (69.3%) and 5187 (58.8%) respectively.

## Measures

### Neighbourhood socioeconomic disadvantage

Each of the 200 neighbourhoods were assigned a socioeconomic score using the ABS’ Index of Relative Socioeconomic Disadvantage (IRSD) [26]. A neighbourhood’s IRSD score reflects each area’s overall level of disadvantage measured based on a number of variables that capture a wide range of socioeconomic attributes, including education, occupation, income, unemployment, household structure, and household tenure (among others). The HABITAT neighbourhoods were grouped into quintiles based on their IRSD scores, with Q1 denoting the 20% least disadvantaged areas relative to the whole of Brisbane, and Q5 the most disadvantaged 20%.

### Self-reported physical function

Physical function was measured using the Physical Function Scale (PF-10), a component of the Short Form 36 Health Survey [27]. The question asks ‘Does your health now limit you in these activities? If so, how much?’. Respondents were given the following response options for each activity: ‘Yes, limited a lot’; ‘Yes, limited a little’; or ‘No, not limited at all’. The PF-10 has been validated among community dwelling older adults [6, 28]. The raw physical function scores were calculated as the sum of re-coded scale items and transformed to a 0-100 scale, where 0 represents minimal functioning and 100 represents maximal functioning. A previous review found a three point difference in PF-10 to be clinically meaningful for effective interventions [29].

### Education

At baseline, respondents were asked to report the highest education qualification they had attained. Consistent with other studies from the HABITAT data [30, 31], education attainment was grouped into four categories: (i) Bachelor degree or higher (included postgraduate diploma, master’s degree, or doctorate), (ii) Diploma (associate or undergraduate), (iii) Vocational (trade or business certificate or apprenticeship), and (iv) No post-secondary school qualification.

### Occupation

At each wave, respondents who were employed at the time of completing the survey were asked to indicate their job title and then to describe the main tasks or duties they performed. The original Australian and New Zealand Standard Classification of Occupations (ANZSCO) classification was used to recode responses into three categories: (i) Manager/professionals, (ii) White-collar employees, and (iii) Blue collar employees. Respondents who were not employed were categorised as follows: (iv) Home duties, (v) Retired or (vi) Permanently unable to work.

### Household income

At each wave, respondents were asked to indicate their total annual household income (including pensions, allowance and investments) using a 14-category measure that was subsequently recoded into six groups: (i) *≥* AU$130,000, (ii) AU$78,800 − 129,999, (iii) AU$52,000–78,799, (iv) AU$26,000–51,999, (v) ≤ AU$25,999, (vi) Not easily classifiable (NEC): Missing/ Don’t know/ Don’t want to answer.

### Neighbourhood self-selection

To assess residential preferences for living in a particular neighbourhood, participants were asked to respond on a five-item Likert scale (ranging from ‘not at all important’ to ‘very important’) to 17 statements asking, ‘How important were the following reasons for choosing your current address?’ Examples of items included: ‘Ease of walking to places’, ‘Closeness to open spaces’ and ‘Closeness to public transport’. Principal Component Analysis with varimax rotation showed that six of the items loaded onto one factor, subsequently described as ‘neighbourhood self-selection’.

### Age and sex

Respondents self-reported their date of birth and sex. The age variable was categorised into five groups: (i) < 50 years, (ii) 50–54 years, (iii) 55–59 years, (iv) 60–64 years, (v) 65 + years and older.

### Statistical analysis

Physical function data were available in waves 4 and 5 (2013 and 2016) therefore, change in physical function over time was observed between these two time points. However, we used data from previous waves to ensure participants in waves 4 and 5 were the same participants who had not moved from wave 1: first, to ensure a consistent neighbourhood environment exposure [32], and second, as relocating to a different neighbourhood may have been influenced by unmeasured preferences related to physical function. The HABITAT baseline (wave 1) sample comprised 11,035 respondents. Of these, 1,916 participants had moved between waves 1 and 5, 5,964 had withdrawn or were non-respondents between waves, while 109 were not the same person between waves, reducing the sample to 3,046 participants. We further excluded those with missing data for exposure, outcome and control variables (*n* = 187). This reduced the analytical sample to 2,859 participants (1,186 men and 1,673 women) from 200 neighbourhoods using complete case analysis.

Longitudinal analyses were undertaken using multilevel linear regression models with three levels: repeated measures over time (level 1), individuals (level 2), and neighbourhoods (level 3). Time-varying variables including physical function scores, neighbourhood self-selection, occupation and household income were modelled at level (1) Individual-level covariates, including age, sex, and education, were modelled at level (2) Neighbourhood-level disadvantage was modelled at level (3). First, we examined the average change in physical function over time (from 2013 to 2016), adjusting for age and sex (Model 1). We then examined change in physical functioning by each socioeconomic indicator. Age and sex were conceptualised as common prior causes (confounders) of the association between education and physical function (Model 2), age, sex and education as confounders of occupation and physical function (Model 3), age, sex, education, and occupation as confounders of household income and physical function (Model 4), and age, sex, all individual-level socioeconomic indicators and neighbourhood self-selection as confounders of the association between neighbourhood disadvantage and physical function (Model 5). These coefficients are presented as pooled differences. Finally, each of the socioeconomic indicators was interacted with time, with coefficients presented as trends over time. Significant differences in trends over time were determined via examining fixed effects and a likelihood ratio test. All models were analysed using STATA/SE 16.0.

## Results

Sociodemographic characteristics and mean physical function scores in 2013 and 2016 are presented in Table 1. There were more men than women, the majority of participants had a bachelor degree or higher, and were managers or professionals, and most participants lived in households earning between $72,800 - $129,999. There were more participants from advantaged neighbourhoods than disadvantaged neighbourhoods. For all participants, physical function was lower in 2016 than in 2013. In both 2013 and 2016, the lowest scores were among those with no post-school qualifications, those who were permanently unable to work, those with a household income less than AU$25,999/year, and those residents living in the most disadvantaged neighbourhoods.

**Table 1:** Physical function scores* by sociodemographic characteristics for the HABITAT sample in 2013 and 2016, *n* = 2,859

	2013		2016	
	%	Physical Function Score, Mean (SD)	%	Physical Function Score, Mean (SD)
**Overall**		85.6 (18.4)		82.6 (19.5)
**Neighbourhood disadvantage**				
Q1 (least disadvantaged)	23.6	90.3 (13.6)	29.0	87.2 (14.3)
Q2	26.2	87.4 (17.3)	22.6	83.6 (18.5)
Q3	21.9	84.9 (18.2)	20.2	82.0 (20.0)
Q4	14.8	83.5 (18.2)	14.8	80.1 (21.0)
Q5 (most disadvantaged)	13.5	77.7 (24.2)	13.4	74.4 (24.7)
**Age (years)**				
< 50	17.8	92.5 (15.2)	5.6	89.4 (14.1)
50–54	21.6	88.3 (16.9)	19.7	86.9 (16.8)
55–59	21.1	86.4 (16.7)	21.7	85.9 (17.2)
60–64	20.2	83.4 (19.5)	20.4	81.3 (19.8)
65–69	19.1	79.6 (21.0)	23.3	78.6 (21.0)
70+	-	-	9.4	74.4 (22.5)
**Sex**				
Men	41.5	88.0 (16.5)	41.5	85.1 (17.3)
Women	58.5	84.0 (19.4)	58.5	80.8 (20.7)
**Education level**				
Bachelor degree or higher	37.7	88.6 (15.8)	37.7	85.9 (17.0)
Diploma	11.7	86.5 (16.8)	11.65	83.0 (19.4)
Vocational	16.9	85.5 (18.8)	16.9	83.7 (17.4)
No post-school qualifications	33.7	82.1 (20.6)	33.7	78.1 (22.1)
**Occupation**				
Manager/professional	33.2	90.2 (13.6)	29.0	88.8 (14.1)
White collar	20.2	88.2 (14.6)	16.0	84.4 (16.7)
Blue collar	9.8	87.9 (17.4)	8.3	86.5 (14.0)
Home duties	4.5	84.1 (19.1)	4.3	80.1 (21.0)
Retired	24.0	80.2 (20.5)	34.7	78.2 (20.7)
Permanently unable to work	1.8	45.7 (30.7)	1.4	42.2 (29.6)
Not easily classifiable	6.2	83.5 (19.3)	6.4	78.6 (24.6)
**Household Income**				
*≥* $130,000	21.3	92.1 (11.8)	19.7	88.9 (14.1)
$72,800 − 129,999	24.8	88.0 (16.3)	23.6	85.5 (17.1)
$41,600 − 72,799	12.2	84.6 (19.7)	12.4	83.5 (18.6)
$26,000–41,599	19.3	80.9 (19.9)	19.0	79.2 (19.9)
< $25,999	10.1	74.7 (23.9)	10.8	71.6 (24.4)
Don’t know/don’t want to say	12.3	87.3 (16.5)	14.6	81.2 (21.0)

*Physical function score ranged from 0-100

Regression coefficients for each pooled model and models examining trends over time are presented in Table 2. The pooled analysis revealed differences in education, with those with no post-school qualifications (β = -4.89, 95%CI -6.44, -3.34) and vocational education levels (β = -1.71, 95%CI -3.23, -0.18) having lower physical function than those with a Bachelor degree or higher. Those who were permanently unable to work (β = -26.57, 95%CI -32.32, -20.83), retired (β = -3.49, 95%CI -4.84, -2.15) and performing home duties (β = -2.84, 95%CI -5.34, -0.34) had lower physical function than managers and professionals. There was a graded association for household income, with lower function among residents of households with income < $25,999 (β = -4.63, 95%CI -6.82, -2.43), $26,000–41,599 (β = -3.23, 95%CI -4.67, -1.78), $41,600 − 72,799 (β = -2.14, 95%CI -3.63, -0.64), and $72,800 − 129,999 (β = -1.48, 95%CI -2.48, -0.47) having lower function compared to those with greater than $130,000. Finally, negative graded associations were observed between the level of neighbourhood disadvantage and physical function. Residents of the Q5 (most disadvantaged) neighbourhoods had, on average, lower function than residents of the most advantaged neighbourhoods (β = -8.16, 95%CI -10.21, -6.12), along with residents of Q4 (β = -4.99, 95%CI -6.68, -3.31) and Q3 neighbourhoods (β = -3.06, 95%CI -4.39, -1.74).

**Table 2:** The associations between neighbourhood disadvantage and individual-level socioeconomic position on physical function from 2013 to 2016

	Pooled differences	Trends over time
	B (95% CI)	B (95% CI)
	**Model 1**	
**Time (ref = 2013)**	-1.57 (-2.17, -0.97)	
**Education level**	**Model 2**	**Model 2a**
Bachelor degree or higher	Ref	Ref
Diploma	-1.56 (-3.40, 0.29)	-0.72 (-2.63, 1.20)
Vocational	-1.71 (-3.23, -0.18)	0.91 (-0.62, 2.45)
No post school qualifications	-4.89 (-6.44, -3.34)	-1.16 (-2.45, 0.12)
Likelihood ratio test		Χ^2^(3) = 7.21, *p* = 0.066
**Occupation**	**Model 3**	**Model 3a**
Manager/professional	Ref	Ref
White collar	-0.33 (-1.51, 0.86)	-1.92 (-3.54, -0.30)
Blue collar	-0.25 (-2.14, 1.64)	-0.48 (-2.90, 1.95)
Home duties	-2.84 (-5.34, -0.34)	-1.61 (-4.61, 1.39)
Retired	-3.49 (-4.84, -2.15)	-0.60 (-2.28, 1.08)
Permanently unable to work	-26.57 (-32.32, -20.83)	1.57 (-5.80, 8.93)
Not easily classifiable	-3.38 (-5.45, -1.32)	-3.91 (-7.38, -0.45)
Likelihood ratio test		Χ^2^(6) = 11.36, *p* = 0.079
**Household Income**	**Model 4**	**Model 4a**
*≥* $130,000	Ref	Ref
$72,800 − 129,999	-1.48 (-2.48, -0.47)	0.01 (-1.54, 1.56)
$41,600 − 72,799	-2.14 (-3.63, -0.64)	1.35 (-0.52, 3.21)
$26,000–41,599	-3.23 (-4.67, -1.78)	0.49 (-1.56, 2.54)
< $25,999	-4.63 (-6.82, -2.43)	-1.11 (-3.52, 1.29)
Don’t know/don’t want to say	-1.23 (-2.58, 0.13)	-1.80 (-3.94, 0.33)
Likelihood ratio test		Χ^2^(5) = 8.00, *p* = 0.156
**Neighbourhood disadvantage**	**Model 5**	**Model 5a**
Q1 (least disadvantage)	Ref	Ref
Q2	-2.05 (-3.03, 1.07)	-0.69 (-2.15, 0.76)
Q3	-3.06 (-4.39, -1.74)	-0.54 (-2.12, 1.02)
Q4	-4.99 (-6.68, -3.31)	0.12 (-1.75, 1.99)
Q5 (most disadvantage)	-8.16 (-10.21, -6.12)	-1.62 (-3.69, 0.44)
Likelihood ratio test		Χ^2^(4) = 3.25, *p* = 0.517

Model 1 = Time adjusted for age and sex

Model 2 = Model 1 + education

Model 2a = Model 2 + education*time

Model 3 = Model 2 + occupation

Model 3a = Model 3 + occupation*time

Model 4 = Model 3 + household income

Model 4a = Model 4a + household income*time

Model 5 = Model 4 + neighbourhood disadvantage

Model 5a = Model 5a + neighbourhood disadvantage*time

When examining trends over time, some individual categories had significantly greater declines in physical function than in the references categories (e.g., white collar workers), however, likelihood ratio tests did not suggest significant differences overall between categories for each of education (*p* = 0.066), occupation (*p* = 0.079), household income (*p* = 0.156) or neighbourhood disadvantage (*p* = 0.517).

## Discussion

This study shows a reduction in physical function from 2013 to 2016 in a cohort of mid-to-older aged Australian adults. Consistent with existing studies [15–17] we found that baseline physical function was lower among those residing in the most disadvantaged neighbourhoods and those with lower educational attainment and lower income. However, we found that, on average, those from disadvantaged neighbourhoods and lower socioeconomic position did not experience physical function decline at a greater rate than those in the reference groups. This finding is at odds with an existing study of Australian women, which found that participants residing in the most disadvantaged neighbourhoods experienced a greater decline in physical function [8]. This discrepancy in findings could be attributed to when the studies were conducted (1998 compared with 2013) and the age of participants at baseline (which ranged from 18 to 75 years). Conversely, our findings are supported by a longitudinal UK study of participants aged 50 years and older (using data from the English Longitudinal Study of Ageing (ELSA) study) that found higher levels of functional limitations in the most deprived neighbourhoods compared to the least deprived neighbourhoods independent of individual socioeconomic indicators [18].

Whilst there is limited evidence of inequalities in decline in physical function between socioeconomic groups, it is important to note that those with lower socioeconomic indicators and those residing in the most disadvantaged neighbourhoods had a lower level of physical function at baseline. This may indicate that exposure to socioeconomic inequalities may influence physical function from a younger age, highlighting the potential need to consider interventions across the life course. The neighbourhood environment has a considerable influence on health and can be an important setting for reducing inequalities in physical function [12]. Increased physical activity may be beneficial in addressing modifiable behaviours that have shown to improve physical function. Creating environments that offer affordable and easily accessible opportunities for physical activity, such as safe, walkable neighbourhoods with pedestrian infrastructure and ample greenspace, may be important for reducing physical function inequalities (33). This type of public health strategy is supported by a World Health Organization (WHO) report promoting ‘ageing in place’ by creating neighbourhoods that facilitate safety, independence, and mobility for older adults [34]. In addition to physical inactivity, lifestyle factors such as poor diet, high body mass index and smoking, have been associated with lower physical functioning [35, 36]. However, individuals from lower socioeconomic positions typically have less exposure to health promoting advice [37]. Therefore, future lifestyle interventions should be tailored to these individuals and consider motivators, such as peer group support, and barriers, such as limited financial resources [37]. Workplaces provide another setting for health promotion and for reducing occupational inequalities in physical function. A recent systematic review by van de Ven, Robroek and Burdorf [38] found evidence that workplace interventions can be more effective at improving health among those from lower socioeconomic backgrounds.

### Strengths and limitations

The large longitudinal sample from socioeconomically diverse neighbourhoods is an important strength of this study. Although physical function data were only available in 2013 and 2016, we were able to include a sample who had remained at the same address since baseline (2007). This allowed the findings to account for temporal dimensions of neighbourhood effects on health as long-term exposure to characteristics of the neighbourhood environment is assumed to have a stronger effect on residents than short-term exposure [32]. Further, neighbourhood self-selection was accounted for in the analyses to minimise self-selection bias [39]. Among the limitations, the data used in this study were from the two latest waves of the HABITAT study and sample attrition from baseline may have implications for the sample generalisability. The likelihood of dropping out between baseline and the included waves tended to be higher for those with lower socioeconomic position, those permanently unable to work and those living in disadvantaged neighbourhoods (data not presented) [22]. If these non-respondents were more likely to have poorer physical function, our findings are likely to underestimate the true magnitude of socioeconomic differences in physical function. The physical function measure was self-reported by participants and may therefore be subject to reporting bias. Some potential confounders, such as ethnicity and Indigenous identify were not measured in this study, while sample attrition also limited the variability of some potentially confounding variables such as country of birth. A difference-in-difference modelling approach, in which within-group comparisons are made and participants effectively serve as their own controls [40], may be a valuable approach for future research, as it also accounts for baseline differences in physical function. Further, although the physical function items have been reported to be sensitive at discriminating low functioning older adults, these items are less sensitive in discerning high functioning older adults [41]. Future studies should incorporate objective performance-based measures, such as the Senior Fitness Test to better understand the complexity of physical function among the ageing population [42]. Finally, relationships between place and health are often context-specific as they are shaped by historical and cultural factors, therefore our study findings may not be generalisable to other states within Australia or other countries.

This study identified socioeconomic inequalities in physical function, and overall physical function decline across a sample of mid-older aged adults. Despite significant decline among the white collar occupation compared to their mangers and professional counterparts, there was little evidence of inequalities in the magnitude of decline in physical function across socioeconomic groups between the two time points.

## Data Availability

Data will be made available on request from the corresponding author.
